# The Prevalence and Associated Factors of Academic Stress among Medical Students of King Khalid University: An Analytical Cross-Sectional Study

**DOI:** 10.3390/healthcare11142029

**Published:** 2023-07-14

**Authors:** Maram M. Al-Shahrani, Bushra S. Alasmri, Reham M. Al-Shahrani, Najwa M. Al-Moalwi, Amar A. Al Qahtani, Aesha F. Siddiqui

**Affiliations:** 1 Saudi Board of Preventive Medicine, Abha Sector, Abha 62583, Saudi Arabia; bualasmari@moh.gov.sa; 2 Family Medicine Specialist, Abha Sector, Abha 62564, Saudi Arabia; remalshahrani@moh.gov.sa; 3 Ministry of Health Saudi Arabia, General of Health Affairs Aseer Region, Al-Areen Primary Health Care Center, Abha 62583, Saudi Arabia; nalmoalwi@moh.gov.sa; 4 College of Medicine, King Khalid University, Abha 61421, Saudi Arabia; ammar_17_17@hotmail.com; 5 Department of Family and Community Medicine, College of Medicine, King Khalid University, Abha 62529, Saudi Arabia; afarheen@kku.edu.sa

**Keywords:** stress, medical students, Abha, Saudi Arabia

## Abstract

Medical students are the category of academic population with the highest levels of stress. This study aimed to assess the prevalence of academic stress among medical students in Saudi Arabia and to identify its associated factors. This cross-sectional study was conducted at the College of Medicine at King Khalid University, Abha. The Medical Student Stressor Questionnaire (MSSQ) was used to evaluate the stress caused by different factors. A total of 422 medical students participated in this study. Among the participants, 115 (27.3%) were male and 307 (72.7%) were female. The highest percentage of students were perceiving moderate to severe stress due to academic-related stressors (97.1%), followed by teaching- and learning-related stressors (93.9%) and group activities-related stressors (88.3%). The lowest domain in which students perceived moderate to severe stress was drive and desire-related stressors (65.8%). The mean percentage of students who perceived moderate-to-severe stress in all domains of stressors was 85.5%. We can conclude that medical students have a high degree of stress, and we emphasize the importance of implementing stress management programs to teach students how to handle stress in order to avoid negative effects on their health and academic performance.

## 1. Introduction

Stress is defined as any form of change leading to physical, emotional, or psycho-logical disturbances [1]. Academic stress is the mental and physical response of the body when academic-related demands are greater than the adaptive abilities of students, especially in the of absence of social support [2]. Academic stress is specifically related to the learning environment; therefore, the measurement scale with which to evaluate this stress is different from the evaluation of general stress. Students consider their time in university as a very stressful period. A study including 2456 students with an average age of 22.5 years reported that academic stress negatively affects critical thinking and academic performance [3].

Medical students represent the most common category of academic populations with high levels of stress [4]. Medical studies especially require students to engage in many stressful activities, and persistent stress causes many adverse effects on students’ mental health. Exams were estimated as high stressors in 63.1% of all students in a study conducted at a medical school in Serbia [5].

Stress is a physiological process that includes endocrine actions, such as autonomic reactions, alterations in cerebrum action, and temperature changes [6]. Jobs or activities with more psychological demands may lead to higher levels of stress. Medical students are under the pressure of high expectations from their teachers, parents, non-medical peers, and sometimes their entire societies [7].

Yusof and Rahim developed the Medical Student Stressor Questionnaire (MSSQ) to recognize the stressors of medical students and measure the intensity of the stress caused by these stressors [8]. Individuals can handle stress to some extent, but their mental and physical conditions can be affected by higher levels of stress [9].

Medical students suffering from stress may have several health problems, such as depression, burnout, low sleep quality, excessive daytime sleepiness, and anxiety [10]. Continuous stress can cause serious diseases, such as strokes, kidney diseases, and heart attacks. However, different individuals perceive similar stressors differently [7]. The university environment is very stressful due to major responsibilities and heavy workloads, especially in the medical field.

A study conducted in southern Saudi Arabia reported that female students and healthcare students were at greater risk of perceiving academic stress. Moreover, high stress was shown to be associated with smoking, lower GPA scores, and insufficient family income [11].

The prevalence of stress among medical students is very high, ranging from 30% to 94% across diverse academic years, from first to final [12,13,14,15]. This high prevalence indicates that stress is considered a huge academic problem.

The prevalence of stress in a study performed in Saudi Arabia in 2019 was 47.4% [16]. The study included 439 medical students at Ibn Sina National College for Medical Studies, Jeddah, Saudi Arabia, during the academic years of 2017–2018. This proportion was inferior to that in other studies performed in Saudi Arabia, which may have been due to continuous improvements in the educational system [17,18].

In Egypt, the prevalence of stress among medical students at Assiut University was reported to be 59.9%, and the female sex was significantly associated with this finding [19].

In a study performed at King Khalid University, 168 participant responses were analyzed, and it was found that a majority of the medical students were moderately stressed (58.34%). The responses on the academic stress scale revealed that exams were the major cause of stress among students. In addition, overall academic stress was significantly positively associated with acne and physical symptoms [20].

Zakirulla et al. reported that female interns and undergraduate students in the clinical years of study at the College of Dentistry at King Khalid University reported higher levels of perceived stress [21].

The estimated prevalences of stress among medical students reported at different universities were different. In addition, there are controversial reports regarding whether males or females are more stressed and which stressor domain has a closer relationship with medical stress. Stress may also extend to the post-graduation period and practical life. Appropriate interventions can help medical students to cope with stress and improve their personal and professional lives.

Therefore, this study aimed to determine the prevalence of stress among medical students of Medicine College at King Khalid University, Abha, Saudi Arabia. We also assessed various demographic factors that lead to stress. The strength of this study is that a stratification of stressors that affect medical students using the MSSQ Questionnaire at King Khalid University has not been conducted previously.

## 2. Materials and Methods

This is an analytical cross-sectional study. This study was conducted from January 2023 to April 2023. The study included male and female students from all academic years at the College of Medicine, King Khalid University, Abha, Saudi Arabia. Students diagnosed with chronic diseases, depression, or anxiety before entering their first year of medical college were excluded from the study.

### 2.1. Sample Size and Sampling Technique

The sample size was estimated by assuming the following factors: margin error = 5%; confidence interval, 95%; and the prevalence of medical stress in medical students who perceived it, 47.4%, as reported by Atwa et al. [16]. The following equation was used, where z is the z-score = 1.96, ε is the margin of error = 0.05, and p is prevalence = 0.474:$n=\frac{z^{2}\times p(1-p)}{\varepsilon^{2}}$.

The minimum required sample size was 383. This number was amplified by 10% to compensate for the non-response rate, yielding a sample size of 422 clinical medical students that was used for this study.

We used the random-sampling technique. Samples were randomly selected from the entire population. Then, we used the random number method to give every student a number using a random number generator. Every student was assigned a number ranging from 1 to 422.

### 2.2. Study Tool and Data Collection

Our study questionnaire was adapted from the Medical Student Stressor Questionnaire (MSSQ), developed by Muhamad Yusof and Ahmad Abdul Rahim to determine the prevalence of stress and domains that highly affect students [8]. It is a valid and reliable instrument for identifying stressors among medical students. For reliability analysis, the total Cronbach’s alpha value was 0.95 [22]

The Medical Student Stressor Questionnaire consists of 40 items. These items were randomly distributed among six domains. Domain (1) was Academic-Related Stressors; domain (2) was Interpersonal and Intrapersonal-Related Stressors; domain (3) was Teaching and Learning-Related Stressors; domain (4) was Social-Related Stressors; domain (5) was Drive and Desire-Related Stressors; and the last domain was Group Activities-Related Stressors. Each item was answered using an assessment scale from 0 to 4; zero referred to no stress at all, one referred to mild stress, two referred to moderate stress, three referred to high stress, and four referred to severe stress. The final calculation from each domain provided us with the ranges of stress levels, as follows: 0–1.00 indicated mild stress, 1.01–2.00 indicated moderate stress, 2.01–3.00 indicated high stress, and 3.01–4.00 indicated severe stress.

The study questionnaire was divided into two parts. The first part consisted of 40 items derived from the MSSQ. The second part included socio-demographic characteristics such as gender, academic year, place of residence (inside or outside Abha), social condition (with or without parents), marital status, having children or not, smoking status, and physical activity.

The questionnaire was randomly distributed (printed or online) to medical students, and they were asked to complete it. Data were collected, checked for errors and completion, and manually entered into a computer for statistical analysis. Data were collected from January 2022 to April 2023, and were handled confidentially and for scientific purposes only.

### 2.3. Statistical Analysis

Descriptive statistics were used to determine the frequency distribution of the study variables. Descriptive analyses are presented as frequencies and percentages for categorical variables. The chi-square test was used to test independence. Bonferroni’s correction was used to assess the relationship between the subsets of each factor. Statistical significance was set at a *p*-value of (<0.05). All collected data were analyzed using SPSS Version 26. Microsoft Excel was also used for domains’ calculations.

## 3. Results

A total of 422 medical students (115 (27.3%) male and 307 (72.7%) female) at King Khalid University, College of Medicine participated in this study. The mean age of participants was 22 years, with a maximum of 28 years and a minimum of 18 years old. All the students experienced some degree of stress. The number and percentage of students with stress in each domain and the mean stress score for each domain are presented in Table 1 and Table 2.

### 3.1. Perceived Stress

**Figure 1:**
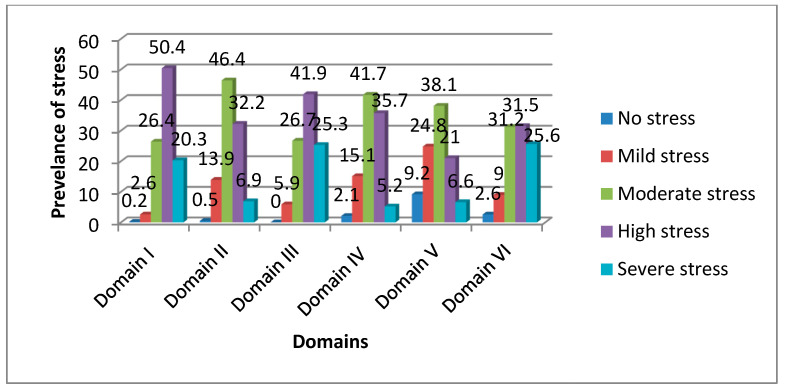
Prevalence and degrees of stress in each domain.

The highest percentage of students were perceiving moderate to severe stress due to academic-related stressors (Domain I, 97.1%). The next most prevalent group was teaching- and learning-related stressors (Domain III, 93.9%), then group activities-related stressors (Domain VI, 88.3%). Most of the students in each domain showed a high degree of stress. The lowest domain in which students perceived moderate-to-severe stress was drive and desire-related stressors (Domain V, 65.8%), and most students in this domain showed a moderate degree of stress. The mean percentage of students who perceived moderate-to-severe stress in all domains was 85.5%. Figure 1 shows the most prevalent degree of stress in each domain.

### 3.2. Stressors

The difference in the degrees of stress in different domains in relation to several social and demographic factors was assessed using the chi-square test, and Bonferroni’s correction was used to assess the relationships between subsets of each factor. We considered no, mild, and moderate stress as no/low stress, and high and severe stress as high stress.

#### 3.2.1. Academic-Related Stressors (Domain I)

There was a significant relationship with gender (*p* = 0.0001), age (*p* = 0.007), place of residence (*p* = 0.001), and smoking status (*p* = 0.021). Female participants were significantly more likely to perceive stress (75.6% vs. 58.3%). Students aged fewer than 20 years old suffered from stress more severely than their older colleagues (87.5 vs. 69%, 58.3%) Students residing outside Abha had higher stress levels (79.6% vs. 65.1%) than those inside Abha. Non-smokers were significantly more likely to perceive stress than ex-smokers (73.4% vs. 51.6%) (see Table 3).

#### 3.2.2. Interpersonal- and Intrapersonal-Related Stressors (Domain II)

There were significant relationships with gender (*p* = 0.0001), age (*p* = 0.024), and social condition (*p* = 0.016). Males had a significantly higher incidence of stress than females (53% vs. 33.9%). Students aged <20 years complained of more stress than those aged 20–25 years (55.4% vs. 36.3%). High stress was more prevalent among students living without a family than those living with a family (53.4% vs. 36.8%) (see Table 4).

#### 3.2.3. Teaching- and Learning-Related Stressors (Domain III)

Regarding teaching and learning stressors, there was a significant relationship with academic year (*p* = 0.011), grade point average (GPA) (*p* = 0.008), and social condition (*p* = 0.04). Third-year students had significantly higher stress levels than internship-year students (100% vs. 75%). Students who had achieved a GPA of 3–4 or >4–5 suffered from higher stress than those with a GPA <3 (95.3%, 94.6 vs. 72.7%). Moreover, students living without their families suffered from higher stress than students living with their families (100% vs. 93.1%) (see Table 5).

#### 3.2.4. Social-Related Stressors (Domain IV)

Gender (*p* = 0.0001), age (*p* = 0.005), academic year (*p* = 0.0001), social condition (*p* = 0.003), and smoking status (*p* = 0.003) were significantly associated with social-related stressors. Male students had significantly higher stress levels than female students (94.8% vs. 78.2%). Students aged <20 years had significantly higher stress levels than their colleagues aged 20–25 years (96.4% vs. 79.8%). Students in their first to fourth years suffered from high stress significantly more often than those in sixth year (97.1%, 90.1%, 92.6%, 92.6% vs. 62.3%). Students living with a family had lower stress levels than those living without a family (80.5 vs. 96.6%). Smoker students perceived higher stress than non-smoker students (97.4% vs. 79.9%) (see Table 6).

#### 3.2.5. Drive- and Desire-Related Stressors (Domain V)

There was a significant relationship with gender (*p* = 0.0001), age (*p* = 0.004), social condition (*p* = 0.004), and smoking status (*p* = 0.009). Males had significantly higher stress levels than females (80.9% vs. 60.3%). Students aged fewer than 20 years had significantly higher stress levels than those aged 20–25 years (83.9 vs. 62.3%). Living without family caused significantly more stress than living with family (82.8% vs. 63.2%). Ex-smokers perceived stress significantly more than non-smokers (87.1% vs. 62.9%) (see Table 7).

#### 3.2.6. Group Activities-Related Stressors (Domain VI)

Regarding domain VI, age (*p* = 0.014) and GPA (*p* = 0.028) had significant relationships with group activities-related stressors. Students younger than 20 years of age suffered from stress significantly more often than those aged 20–25 or >25 years (100% vs. 86.5%, 87.5%, respectively). Students scoring < 3 on their GPAs had lower stress levels than students scoring 3–4 or >4–5 (63.6% vs. 90.7%, 88.6%) (see Table 8).

## 4. Discussion

Many significant factors can affect academic performance, such as fear of delay, student engagement, parental support, teacher support, facilitating conditions, and stress levels [23].

Students’ stress is an important reason for their low study performance. Chronic exposure to stressful conditions negatively affects students’ emotional, mental, and physical well-being. Medical students are the category of the academic population with the highest levels of stress [4]. Persistent stressful conditions are associated with various health problems in medical students at different stages of their learning [24].

Students experience several stressors during academic learning. These may be academic, interpersonal and intrapersonal, teaching- and learning-related, social, drive- and desire-related, and group activities-related stressors, as was mentioned by Yusoff and Rahim [22].

The prevalence of moderate-to-severe stress in medical students of all years in our setting was 85.5%, which is comparable to other studies conducted in different countries. We found that academic- (97.1%), teaching- and learning- (93.9%), and group activities-related stressors (88.3%) affected the most students and scored the highest in terms of degrees of stress. Academic stressors were related to examinations, marks, and grading methods. Therefore, students perceived high levels of stress in this domain. Moreover, teaching- and learning-related stressors included tasks and feedback given to students, as well as the quality of the relationships between teachers and their students, as any disturbance in this relationship or unfriendly relationship may cause stress for students. Most medical activities involve working in groups. Therefore, if students have difficulties with group activities, they may be easily distressed.

A similar study from Syria reported that the prevalence of stress among medical students was 87.6%, and academic-related stressors were the most important in terms of perceiving stress [25]. Furthermore, academic-related problems were the most significant factors found to be associated with stress in a Malaysian university [26]. In accordance with our study, Geroge and Joseph found that 85% of students experienced stress [27]. Another study from India also showed that 85% of students underwent stress [28]. Moreover, in a study conducted in a medical college, Surat reported that nearly 97% of students experienced mild-to-severe stress [29].

In contrast, a single-center study in India found that the prevalence of stress was 38%. The most highly scored stressors were academic- (62%), social- (46%), teaching- and learning- (31%), and group activities-related stressors (35%). These percentages seem to be lower than our findings, which may be because the study was conducted on only first-year students and used a smaller sample size (only 150 students) than our study [15].

Our study revealed that female students were more stressed than their male counterparts in terms of academic-related stressors. However, male students perceived more stress in interpersonal and intrapersonal, social-related, and drive- and desire-related stressors. This may be due to female emotional characteristics and attitudes towards academic situations. Deatherage et al. are in agreement with our findings [30]. Furthermore, stress was significantly higher among female medical students than among their male counterparts in Jeddah, Saudi Arabia. Poor friend support was the only significant factor for high stress among the female students in this study [31]. According to Backović et al., academic- and exam-related stressors were more frequent among female students. Female students frequently reported highly stressful effects of contact with patients and conducting autopsies [32].

A study conducted in Sudan also agreed with our finding that female medical students were more stressed due to academic-related stressors than male students [33].

Our study found that males perceived high stress scores in interpersonal and intrapersonal, social-, and drive- and desire-related stressors. This may be because they were unfamiliar with social relations. Furthermore, drive- and desire-related stressors are associated with an unwillingness to study, and males tend to be more interested in clinical work than studying. In agreement with our results, male students were also found to be more stressed than female students in study conducted at a dental college [34].

A cross-sectional study conducted in Indonesia disagreed with this study, reporting that females are more stressed than males in terms of drive- and desire-related stressors [35].

Another dissimilar finding was found at the Mansoura College of Medicine, Egypt. There was no significant difference between males and females in their perception of stress [14].

We found that students under 20 years of age perceived higher levels of stress than others in most domains. This can be explained by the fact that older students have diverse strategies with which to handle stress [36]. A similar finding was reported by Onolemhenhen, Ebiega Enokela [37]. However, there was no significant relationship between age and perceived stress found in a Saudi study [12].

Our study revealed that sixth-year students had lower stress levels than students in lower grades in terms of social-related stressors. This may be because sixth-year students become familiar with college and social relationships. Third-year students are more stressed than internship students in term of teaching- and learning-related stressors, because internship students are more interested in clinical work and understand how to learn and overcome teaching-related problems.

In agreement with our study, a study from India showed that first-year medical students had a higher prevalence of stress, and the most important stressors were academic stressors. Difficulty in understanding the content, a large amount of content, and a shortage of time with which to revise the subjects were the main factors leading to academic stress [7]. Moreover, according to a study conducted in Nigeria, academic stress increases in first-year students, declines in their second and third years, and rises again in their final year. Stress may rise again in the final year due to an overload of clinical work [37].

Another study disagreed with our findings, reporting that first-year medical students had lower stress levels than final-year medical students and that choosing to study medicine allows students to handle perceived stress well [33].

Our study reported that students with GPA scores lower than 3 perceived lower stress levels than those scoring higher in terms of teaching- and learning-related stressors. This can be explained by the fact that they are carless with studying and not interested in achieving high scores, while students achieving high scores are stressed to continuously remain at the same level. Dissimilar findings to our results from a study conducted among pharmacy students in Malaya reported that there was a negative correlation between GPA and stress level because a certain amount of stress is required for better academic performance [38]. A study conducted at the Caribbean Medical School showed no correlation between stress and GPA [39].

Residing outside Abha caused students to suffer from academic related-stress. In a study conducted in Damascus, students who lived outside Damascus suffered from more stress than those who lived inside Damascus in terms of teaching-, learning-, and social-related stressors. However, living inside Damascus caused students to perceive more moderate stress regarding academic-, and interpersonal-, and intrapersonal-related stressors [25]. Residence outside the university’s city causes students to face transportation pressure and take more time to arrive at their college; thus, they become more tired and stressed due to arriving late. A lack of time to study was also considered as a cause for stress perception.

We noticed that students who lived without family experienced more stress in most of the stressor categories because of a lack of family support. Parental support significantly reduced students’ stress levels, as was reported by Wijaya et al. [23]. In disagreement with our results, there was no difference in stress between medical students who lived with and without their families in a study conducted in Indonesia [35].

In the present study, smokers perceived higher stress levels than non-smokers in terms of social-related stressors. This is because smokers become more socially separated and lonely than non-smokers [40]. Similar results were found in central Iran; smokers had higher stress levels than non-smokers and ex-smokers [41].

Moreover, non-smokers perceived academic-related stress more significantly than ex-smokers. Ex-smokers have more self-confidence and happier feelings after ceasing smoking, so they perceived lower stress levels. However, ex-smokers had higher stress levels regarding drive- and desire-related stressors.

Physical activity can be used as a strategy for managing stress and preventing the activation of different inflammatory pathways that lead to various metabolic, cardiovascular, and mental diseases [42]. However, in our study, physical activity had no significant relationship with the perception of stress. Our study has its strengths and limitations. This study included all academic years of medicine college. Furthermore, our study was not restricted to a specific stressor; instead, we evaluated six possible domains of stress.

The main limitation of this study is that it was conducted at one college at one university. Moreover, the small sample size is considered a limitation of this study. More studies are needed which include other medical colleges, such as pharmacy and dental colleges, in different universities, as well as larger sample sizes.

## 5. Conclusions

The prevalence of stress is high among medical students, and academic stressors are the most important cause of stress in medical students. Females are more stressed than their male colleagues in terms of academic-related stressors. Males suffer from more stress than females in social-, drive- and desire-, and interpersonal- and intrapersonal-related stressors. The perception of stress in our study decreased with age. Living outside Abha and without a family was shown to have a significant role in the perception of severe stress among medical students. We recommend initiating stress management programs to teach students how to handle stress during their medical education.

## Figures and Tables

**Table 1:** Degree of stress and mean stress score across all six domains.

	No Stress N (%)	Mild Stress N (%)	Moderate Stress N (%)	High Stress N (%)	Severe Stress N (%)	Total Percentage of Stressed Students (Moderate–Severe)
Domain I	1 (0.2)	11 (2.6)	111 (26.4)	213 (50.4)	86 (20.3)	410 (97.1%)
Domain II	2 (0.5)	59 (13.9)	196 (46.4)	136 (32.2)	29 (6.9)	361 (85.5%)
Domain III	0 (0)	25 (5.9)	113 (26.7)	177 (41.9)	107 (25.3)	397 (93.9%)
Domain IV	9 (2.1)	64 (15.1)	176 (41.7)	151 (35.7)	22 (5.2)	349 (82.7%)
Domain V	39 (9.2)	105 (24.8)	161 (38.1)	89 (21)	28 (6.6)	278 (65.8%)
Domain VI	11 (2.6)	38 (9)	132 (31.2)	133 (31.5)	108 (25.6)	373 (88.3%)
Mean	10 (2.4)	50 (12)	148 (35)	150 (35.5)	63 (15)	361 (85.5%)

**Table 2:** Mean stress score for each domain.

	Mean Score
Domain I	2.42 (High stress)
Domain II	1.86 (Moderate stress)
Domain III	2.45 (High stress)
Domain IV	1.89 (Moderate stress)
Domain V	1.59 (Moderate stress)
Domain VI	2.34 (High stress)

**Table 3:** Relationships between different factors and Domain I (academic-related stressors).

Items	No/Low Stress N (%)	High Stress N (%)	*p*-Value
Gender			
Male	48 (41.7) *	67 (58.3)	0.0001
Female	75 (24.4)	232 (75.6) *	
Age			
<20	7 (12.5)	49 (87.5) *	0.007
20–25	106 (31) *	236 (69)	
>25	10 (41.7) *	14 (58.3)	
Academic Year			
First	4 (11.8)	30 (88.2)	0.018 #
Second	14 (19.7)	57 (80.3)	
Third	23 (28.4)	58 (71.6)	
Fourth	15 (27.8)	39 (72.2)	
Fifth	19 (34.5)	36 (65.5)	
Sixth	45 (36.9)	77 (63.1)	
Internship	3 (75)	1 (25)	
Graduated	0	1 (100)	
GPA			
<3	6 (54.5)	5 (45.5)	0.071
3–4	25 (23.4)	82 (76.6)	
>4–5	89 (30)	208 (70)	
Place of residence			
Inside Abha	89 (34.9) *	166 (65.1)	0.001
Outside Abha	34 (20.4)	133 (79.6) *	
Social condition			
Live with family	105 (28.8)	259 (71.2)	0.733
Live without family	18 (31)	40 (69)	
Marital status	0		
Single	122 (30.4)	279 (69.6)	0.094
Married	1(5.3)	18 (94.7)	
Divorced	0	1 (100)	
Widowed	0	1 (100)	
Do you have children			
Yes	0	5 (100)	0.149
No	123 (29.5)	294 (70.5)	
Smoking status			
Smoker	14 (36.8)	24 (63.2)	0.021
Non-smoker	94 (26.6)	259 (73.4) *	
Ex-smoker	15 (48.4) *	16 (51.6)	
Physical activity			
Yes	52 (28.4)	131(71.6)	0.772
No	71 (29.7)	168 (70.3)	
Physical activity (minutes/day)			
15–59	34 (30.4)	78 (69.6)	0.335
60–120	15 (25.9)	43 (74.1)	
>120	0	7 (100)	

GPA: Grade point average. * indicates a more significant difference than other groups in the same category. # indicates that Bonferroni’s correction revealed no significant difference in the perception of stress between groups.

**Table 4:** Relationships between different factors and Domain II (Interpersonal- and intrapersonal-related stressors).

Items	No/Low Stress N (%)	High Stress N (%)	*p*-Value
Gender			
Male	54 (47)	61 (53) *	0.0001
Female	203(66.1) *	104 (33.9)	
Age			
<20	25 (44.6)	31 (55.4) *	0.024
20–25	218 (63.7) *	124 (36.3)	
>25	14 (58.3)	10 (41.7)	
Academic Year			
First	21 (61.8)	13 (38.2)	0.021 #
Second	36 (50.7)	35 (49.3)	
Third	54 (66.7)	27 (33.3)	
Fourth	25 (46.3)	29 (53.7)	
Fifth	33 (60)	22 (40)	
Sixth	84 (68.9)	38 (31.1)	
Internship	4 (100)	0	
Graduated	0	1 (100)	
GPA			
<3	8 (72.7)	3 (27.3)	0.358
3–4	70 (65.4)	37 (34.6)	
>4–5	175 (58.9)	122 (41.1)	
Place of residence			
Inside Abha	151 (59.2)	104 (40.8)	0.381
Outside Abha	106 (63.5)	61 (36.5)	
Social condition			
Live with family	230 (63.2) *	134 (36.8)	0.016
Live without family	27 (46.6)	31 (53.4) *	
Marital status			
Single	243 (60.6)	158 (39.4)	0.446
Married	13 (68.4)	6 (31.6)	
Divorced	1 (100)	0	
Widowed	0	1 (100)	
Do you have children			
Yes	4 (80)	1 (20)	0.379
No	253 (60.7)	164 (39.3)	
Smoking status			
Smoker	17 (44.7)	21 (55.3)	0.012 #
Non-smoker	226 (64)	127 (36)	
Ex-smoker	14 (45.2)	17 (54.8)	
Physical activity			
Yes	109 (59.6)	74 (40.4)	0.622
No	148 (61.9)	91 (38.1)	
Physical activity (minutes/day)			
15–59	71(63.4)	41 (36.6)	0.231
60–120	32 (55.2)	26 (44.8)	
>120	2 (28.6)	5 (71.4)	

GPA: Grade point average. * indicates a higher significant difference than other groups in the same category. # indicates that Bonferroni’s correction revealed no significant difference in the perception of stress between groups.

**Table 5:** Relationships between different factors and Domain III (Teaching- and learning-related stressors).

Items	No/Low Stress N (%)	High Stress N (%)	*p*-Value
Gender			
Male	4 (3.5)	111 (96.5)	0.193
Female	21 (6.8)	286 (93.2)	
Age			
<20	1 (1.8)	55 (98.2)	0.325
20–25	23 (6.7)	319 (93.3)	
>25	1 (4.2)	23 (95.8)	
Academic Year			
First	0	34 (100)	0.011
Second	3 (4.2)	68 (95.8)	
Third	0	81 (100) *	
Fourth	2 (3.7)	52 (96.3)	
Fifth	6 (10.9)	49 (89.1)	
Sixth	13 (10.7)	109 (89.3)	
Internship	1 (25) *	3 (75)	
Graduated	0	1 (100)	
GPA			
<3	3 (27.3) *	8 (72.7)	0.008
3–4	5 (4.7)	102 (95.3) *	
>4–5	16 (5.4)	281 (94.6) *	
Place of residence			
Inside Abha	16 (6.3)	239 (93.7)	0.706
Outside Abha	9 (5.4)	158 (94.6)	
Social condition			
Live with family	25 (6.9) *	339 (93.1)	0.04
Live without family	0	58 (100) *	
Marital status			
Single	25 (6.2)	376 (93.8)	0.707
Married	0	19 (100)	
Divorced	0	1 (100)	
Widowed	0	1 (100)	
Do you have children			
Yes	0	5 (100)	0.572
No	25 (6)	392 (94)	
Smoking status			
Smoker	2 (5.3)	36 (94.7)	0.781
Non-smoker	22 (6.2)	331 (93.8)	
Ex-smoker	1 (3.2)	30 (96.8)	
Physical activity			
Yes	10 (5.5)	173 (94.5)	0.726
No	15 (6.3)	224 (93.7)	
Physical activity (minutes/day)			
15–59	10 (8.9)	102 (91.1)	0.115
60–120	0	58 (100)	
>120	0	7 (100)	

GPA: Grade point average. * indicates a more significant difference than other groups in the same category.

**Table 6:** Relationships between different factors and Domain IV (social-related stressors).

Items	No/Low Stress N (%)	High Stress N (%)	*p*-Value
Gender			
Male	6 (5.2)	109 (94.8) *	0.0001
Female	67 (21.8) *	240 (78.2)	
Age			
<20	2 (3.6)	54 (96.4) *	0.005
20–25	69 (20.2)*	27 (79.8)	
>25	2 (8.3)	22 (91.7)	
Academic Year			
First	1 (2.9)	33 (97.1) *	0.0001
Second	7 (9.9)	64 (90.1) *	
Third	6 (7.4)	75 (92.6) *	
Fourth	4 (7.4)	50 (92.6) *	
Fifth	8 (14.5)	47 (85.5)	
Sixth	46 (37.7) *	76 (62.3)	
Internship	1 (25)	3 (75)	
Graduated	0	1 (100)	
GPA			
<3	4 (36.4)	7 (63.6)	0.230
3–4	19 (17.8)	88 (82.2)	
>4–5	49 (16.5)	248 (83.5)	
Place of residence			
Inside Abha	48 (18.8)	207 (81.2)	0.306
Outside Abha	25 (15)	142 (85)	
Social condition			
Live with family	71 (19.5) *	293 (80.5)	0.003
Live without family	2 (3.4)	56 (96.6) *	
Marital status			
Single	72 (18)	329 (82)	0.482
Married	1 (5.3)	18 (94.7)	
Divorced	0	1 (100)	
Widowed	0	1 (100)	
Do you have children			
Yes	0	5 (100)	0.304
No	73 (17.5)	344 (82.5)	
Smoking status			
Smoker	1 (2.6)	37 (97.4) *	0.003
Non-smoker	71 (20.1) *	282 (79.9)	
Ex-smoker	1 (3.2)	30 (96.8)	
Physical activity			
Yes	27 (14.8)	156 (85.2)	0.227
No	46 (19.2)	193 (80.8)	
Physical activity (minutes/day)			
15–59	14 (12.5)	98 (87.5)	0.406
60–120	11 (19)	47 (81)	
>120	2 (28.6)	5 (71.4)	

GPA: Grade point average. * indicates a more significant difference than other groups in the same category.

**Table 7:** Relationships between different factors and Domain V (Drive- and desire-related stressors).

Items	No/Low Stress N (%)	High Stress N (%)	*p*-Value
Gender			
Male	22 (19.1)	93 (80.9) *	0.0001
Female	122 (39.7) *	185 (60.3)	
Age			
<20	9 (16.1)	47 (83.9) *	0.004
20–25	129 (37.7) *	213 (62.3)	
>25	6 (25)	18 (75)	
Academic Year			
First	6 (17.6)	28 (82.4)	0.002 #
Second	15 (21.1)	56 (78.9)	
Third	35 (43.2)	46 (56.8)	
Fourth	16 (29.6)	38 (70.4)	
Fifth	16 (29.1)	39 (70.9)	
Sixth	52 (42.6)	70 (57.4)	
Internship	3 (75)	1 (25)	
Graduated	1 (100)	0	
GPA			
<3	4 (36.4)	7 (63.6)	0.414
3–4	31 (29)	76 (71)	
>4–5	107 (36)	190 (64)	
Place of residence			
Inside Abha	88 (34.5)	167 (65.5)	0.836
Outside Abha	56 (33.5)	111 (66.5)	
Social condition			
Live with family	134 (36.8) *	230 (63.2)	0.004
Live without family	10 (17.2)	48 (82.8) *	
Marital status			
Single	141 (35.2)	260 (64.8)	0.254
Married	3 (15.8)	16 (84.2)	
Divorced	0	1 (100)	
Widowed	0	1 (100)	
Do you have children			
Yes	0	5 (100)	0.105
No	144 (34.5)	273 (65.5)	
Smoking status			
Smoker	9 (23.7)	29 (76.3)	0.009
Non-smoker	131 (37.1) *	222 (62.9)	
Ex-smoker	4 (12.9)	27 (87.1) *	
Physical activity			
Yes	57 (31.1)	126 (68.9)	0.259
No	87 (36.4)	152 (63.6)	
Physical activity (minutes/day)			
15–59	39 (34.8)	73 (65.2)	0.440
60–120	16 (27.6)	42 (72.4)	
>120	1 (14.3)	6 (85.7)	

GPA: Grade point average. * indicates a more significant difference than other groups in the same category. # indicates that Bonferroni’s correction revealed no significant differences in the perception of stress between groups.

**Table 8:** Relationships between different factors and Domain VI (group activities-related stressors).

Items	No/Low Stress N (%)	High Stress N (%)	*p*-Value
Gender			
Male	9 (7.8)	106 (92.2)	0.137
Female	40 (13)	267 (87)	
Age			
<20	0	56 (100) *	0.014
20–25	46 (13.5) *	296 (86.5)	
>25	3 (12.5) *	21 (87.5)	
Academic Year			
First	0	34 (100)	0.003 #
Second	5 (7)	66 (93)	
Third	5 (6.2)	76 (93.8)	
Fourth	4 (7.4)	50 (92.6)	
Fifth	8 (14.5)	47 (85.5)	
Sixth	26 (21.3)	96 (78.7)	
Internship	1 (25)	3 (75)	
Graduated	0	1 (100)	
GPA			
<3	4 (36.4) *	7 (63.6)	0.028
3–4	10 (9.3)	97 (90.7) *	
>4–5	34 (11.4)	263 (88.6) *	
Place of residence			
Inside Abha	33 (12.9)	222 (87.1)	0.292
Outside Abha	16 (9.6)	151 (90.4)	
Social condition			
Live with family	46 (12.6)	318 (87.4)	0.099
Live without family	3 (5.2)	55 (94.8)	
Marital status			
Single	49 (12.2)	352 (87.8)	0.407
Married	0	19 (100)	
Divorced	0	1 (100)	
Widowed	0	1 (100)	
Do you have children			
Yes	0	5 (100)	0.415
No	49 (11.8)	386 (88.2)	
Smoking status			
Smoker	3 (7.9)	35 (92.1)	0.214
Non-smoker	45 (12.7)	308 (87.3)	
Ex-smoker	1 (3.2)	30 (96.8)	
Physical activity			
Yes	22 (12)	161 (88)	0.818
No	27 (11.3)	212 (88.7)	
Physical activity (minutes/day)			
15–59	17 (15.2)	96 (84.8)	0.408
60–120	5 (8.6)	53 (91.4)	
>120	0	7 (100)	

GPA: Grade point average. * indicates a more significant difference than other groups in the same category. # indicates that Bonferroni’s correction revealed no significant differences in the perception of stress between groups.

## Data Availability

All data generated or analyzed during this study are included in this **p**ublished article.
